# Serum 20S proteasome is elevated in patients with renal cell carcinoma and associated with poor prognosis

**DOI:** 10.1038/bjc.2012.20

**Published:** 2012-01-31

**Authors:** M de Martino, K Hoetzenecker, H J Ankersmit, G A Roth, A Haitel, M Waldert, T Klatte

**Affiliations:** 1 Department of Urology, Medical University of Vienna, Währinger Gürtel 18–20, Vienna 1090, Austria; 2 Department of Thoracic Surgery, Medical University of Vienna, Währinger Gürtel 18–20, Vienna 1090, Austria; 3 Department of Anesthesiology, General Intensive Care and Pain Medicine, Medical University of Vienna, Währinger Gürtel 18–20, Vienna 1090, Austria; 4 Department of Clinical Pathology, Medical University of Vienna, Währinger Gürtel 18–20, Vienna 1090, Austria

**Keywords:** 20S proteasome, renal cancer, hypoxia, diagnosis, prognosis

## Abstract

**Background:**

To date, no reliable serum marker for clear cell renal cell carcinoma (CCRCC) is available. The aim of this study was to evaluate the putative significance of circulating 20S proteasome levels.

**Methods:**

Preoperative 20S proteasome serum levels were determined in 113 CCRCC patients and 15 healthy controls by a sandwich enzyme-linked immunosorbent assay. Associations with CCRCC, pathological variables, disease-specific survival (DSS), and response to sunitinib were evaluated.

**Results:**

Median 20S proteasome levels were higher in CCRCC patients than in healthy controls (4.66 *vs* 1.52 *μ*g ml^−1^, *P*<0.0001). The area under the receiver operating characteristics curve curve was 87.1%. The 20S proteasome levels were associated with symptoms (*P*=0.0008), distant metastases (*P*=0.0011), grade (*P*=0.0247), and necrosis (*P*=0.0462). The 20S proteasome levels were identified as a prognostic factor for DSS in both univariable (hazards ratio 1.21, *P*<0.001) and multivariable (hazards ratio 1.17, *P*=0.0015) survival analysis. In patients responding to sunitinib, 20S proteasome levels were lower than in patients with stable disease and progressive disease.

**Conclusion:**

This study demonstrates for the first time that increased 20S proteasome levels are associated with CCRCC, advanced disease, and poor prognosis. Routine use of this marker may allow better diagnosis, risk stratification, risk-adjusted follow-up, and identification of patients with a greater likelihood of response to targeted therapy.

More than 250 000 patients are newly diagnosed with renal cell carcinoma (RCC) worldwide annually (Ferlay *et al*, 2010). Although the mortality rate has stabilised or declined in most countries since the 1990s, more than 100 000 deaths are attributed to the disease every year (Ferlay *et al*, 2010).

During the last decades, our understanding of RCC biology has improved considerably. Several subtypes have been delineated, including clear cell RCC (CCRCC), papillary, chromophobe, collecting duct, and unclassified RCC. Additionally, subtype-specific molecular pathways and the genetic basis of these pathways have been characterised (Klatte and Pantuck, 2008). This knowledge led to the development of novel systemic therapies that have changed the therapeutic landscape (Gore and Larkin, 2011; Powles *et al*, 2011; Procopio *et al*, 2011). Research showed that the biology of CCRCC is in part driven by the hypoxia-inducible factor (HIF) protein family, the *von Hippel-Lindau* (*VHL*) gene, and the ubiquitin-proteasome pathway (Klatte and Pantuck, 2008). HIF serves as a transcription factor of hypoxia-inducible genes such as *vascular endothelial growth factor* (*VEGF*), *platelet-derived growth factor*, *transforming growth factor-α*, *carbonic anhydrase IX*, and the chemokine receptor CXCR4 (Klatte and Pantuck, 2008). HIF and its downstream targets have been identified as key regulators of CCRCC progression (Klatte *et al*, 2007b; Klatte and Pantuck, 2008).

Under conditions of normoxia and wild-type *VHL*, HIF is labelled with ubiquitin and subsequently degraded via the proteasome (Corn, 2007). In CCRCC, the ubiquitin–proteasome pathway is frequently impacted due to hypoxia and *VHL* loss. A constitutively increased proteasome activity has been observed in CCRCC, which correlates with proliferation (Kanayama *et al*, 1991). Despite HIF, peptides and proteins involved in basic cellular processes, such as cell cycle regulation, apoptosis, signal transduction, transcriptional activation, antigen processing, and cancer cachexia are processed via the proteasome (Tisdale, 2003; Roth *et al*, 2005). This multi-catalytic protease complex is located in both the cytoplasm and the nucleus. Its 20S subunit constitutes the core of the proteasome. Each core of a 20S subunit is further assembled by the 19S regulatory subunit, which together form the 26S proteasome. Upon translocation of ubiquitinated proteins into the proteasome, the target protein undergoes ATP-dependent degradation (Voges *et al*, 1999). Importantly, function of the 20S proteasome can be reversibly inhibited by bortezomib (Groll *et al*, 2006). In metastatic RCC, clinical responses have been observed following administration of this agent (Kondagunta *et al*, 2004).

To date, no reliable diagnostic or prognostic serum marker for CCRCC is available. As 20S proteasome levels can be detected in the serum, it may represent a novel biomarker for this entity, which could facilitate diagnosis and postoperative risk-group assessment, and may help in identifying patients that most likely respond to targeted agents. To date, however, no data have been reported. In the present pilot study, we have analysed preoperative 20S proteasome serum levels in CCRCC patients and healthy controls, using a validated enzyme-linked immunosorbent assay (Roth *et al*, 2005; Szerafin *et al*, 2008). Further, we have investigated the association of 20S proteasome serum levels with clinical and pathological factors, response to targeted agents, and survival, to determine its potential predictive and prognostic significance.

## Patients and methods

### Study population

This study was reviewed and approved by the institutional review board of the Medical University of Vienna, Austria. A total of 128 peripheral venous blood samples (113 with CCRCC, 15 healthy blood donors) were obtained at the Department of Urology, Medical University of Vienna, Austria, between February 2008 and July 2009. To minimise protein degradation, the samples were processed immediately. Following upright storage for 10 min, serum was obtained after centrifugation (1800 **g**, 10 min) and stored at −80 °C until analysis. The 113 CCRCC serum samples were selected out of 153 preoperative serum samples, which were collected from consecutive patients with an RCC-suspicious renal tumour scheduled for partial or radical nephrectomy. The excluded 40 serum samples refer to patients with non-CCRCC (*n*=22), bilateral RCC (*n*=2), hereditary RCC (*n*=1), or benign renal tumours (*n*=15).

Baseline clinical and pathological data were abstracted from a prospectively maintained institutional review board-approved kidney cancer database, and included age, gender, symptoms, 2009 TNM stage (Sobin *et al*, 2009), Fuhrman grade, pathological tumour size, and tumour necrosis. Diagnosis of CCRCC, the Fuhrman grade, tumour necrosis, and TNM stage was confirmed by one dedicated uro-pathologist (AH).

Radical and partial nephrectomy was performed in 59 (52%) and 54 (48%) patients, respectively. None of the patients received adjuvant therapy. All patients with non-metastatic disease were followed systematically at our outpatient clinic according to the established guidelines (Ljungberg *et al*, 2007). Of the 32 patients who developed or presented with metastatic disease, the first-line therapy comprised sunitinib (50 mg po q24h, *n*=22), temsirolimus (25 mg iv q7d, *n*=2), metastasectomy (*n*=3), or best supportive care (*n*=5). In patients receiving sunitinib and temsirolimus, imaging was repeated after two cycles and in 8-week-intervals, respectively. Response was evaluated according to the RECIST criteria. Median follow-up for the patients alive was 30 months (interquartile range, 14 months). Out of 25 patients, who were deceased at last follow-up, 21 died from CCRCC.

### Serum 20S proteasome measurements

The 20S proteasome serum levels were determined by a sandwich enzyme-linked immunosorbent assay (Roth *et al*, 2005; Szerafin *et al*, 2008). Microtitration plates were coated overnight with the mouse monoclonal antibody to the 20S proteasome subunit a6 (1 : 4500 in carbonate buffer, pH 9.6, Affiniti Research Products Ltd, Exeter, UK). Remaining binding sites were blocked with 0.5% fetal calf serum in PBS, pH 7.4. Serum samples were diluted 1 : 20 and applied to each well for 3 h at room temperature. Standard curves were established using 20S proteasome (Affiniti Research Products Ltd) in a concentration from 5000 to 78 ng ml^−1^ (Affiniti Research Products Ltd). After a washing step, a rabbit polyclonal antibody to 20S proteasome *α*/*β*-subunits was added for 2 h at room temperature (Affiniti Research Products Ltd). Following another washing step, a peroxidase-conjugated mouse anti-rabbit IgG (Jackson ImmunoResearch, West Grove, PA, USA) was used for the detection of the antigen. The bound antibodies were detected with tetramethylbenzidine as substrate. The reaction was stopped with sulphuric acid and OD values were determined at 450 nm. To exclude the possibility of non-specific binding, we tested bovine serum albumin as control protein instead of 20S proteasome, and did not observe any positive reaction.

### Statistical analysis

The continuous data were tested for normal distribution using the Kolmogorov–Smirnov test and were found to be not normally distributed. Thus, associations of 20S proteasome serum levels with clinical and pathological parameters were assessed with non-parametric Kruskal–Wallis rank sum tests. Correlations were determined using the non-parametric Spearman's rank correlation. The diagnostic performance of 20S proteasome serum levels was analysed with a receiver operating characteristics curve (ROC). The ROC curve is a plot of sensitivity *vs* 1-specificity for all possible cut-point values. The area under the ROC curve was applied to evaluate the diagnostic accuracy. The 95% confidence intervals (95% CI) were calculated according to the DeLong method.

To assess 20S proteasome serum levels as a prognostic marker, associations with disease-specific survival (DSS) time were assessed. DSS was calculated from the date of surgery. A cut point for prognostic sub-stratification was identified with recursive partitioning-based survival tree analysis. Univariable and multivariable Cox proportional hazards models were fit to evaluate the relative impact of variables on DSS. Because of the low number of events, an over-fit bias was likely to occur in multivariable analysis. Thus, not all relevant prognostic factors were included as a single variable. Rather, the main prognostic factors (T, N, M stage, size, grade, and necrosis) were combined as SSIGN score (Frank *et al*, 2002). Predictive accuracies were assessed by concordance index. The likelihood ratio test was applied to compare predictive accuracies between nested models. Statistical analyses were all performed with the freely available statistical package R-2.10.1 (http://cran.r-project.org/) using the Epi, Design and survival libraries. A *P*-value <0.05 was considered statistically significant.

## Results

### Association with CCRCC

**Figure 1:**
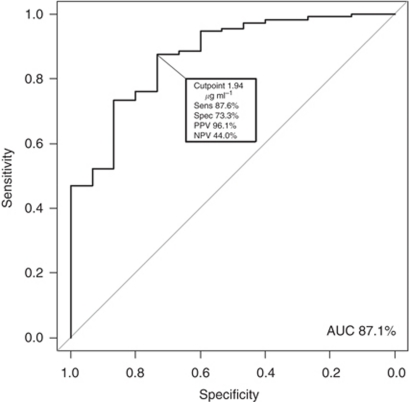
Accuracy of 20S proteasome serum levels for diagnosis of CCRCC. The area under the ROC curve (AUC) was 87.1%. A cut point of 1.94 *μ*g ml^−1^ showed the highest diagnostic accuracy.

Overall median serum level of 20S proteasome was 4.21 *μ*g ml^−1^ (interquartile range, 4.29), and the levels differed significantly between the CCRCC patients (median 4.66 *μ*g ml^−1^) and healthy controls (1.52 *μ*g ml^−1^, *P*<0.0001). The area under the ROC curve under the ROC curve was 87.1% (95% CI, 77.8–96.5). At the optimal cut point, the sensitivity and the specificity reached 87.6% (95% CI, 80.1–93.1) and 73.3% (95% CI, 44.9–92.0), respectively (Figure 1).

### Association with clinical and pathological variables

Characteristics of CCRCC patients and associations with 20S proteasome serum levels are shown in Table 1. The 20S proteasome serum levels were higher in patients with CCRCC-associated symptoms (*P*=0.0008), with distant metastases (*P*=0.0011), higher Fuhrman grades (*P*=0.0247), and necrotic tumours (*P*=0.0462). Additionally, there was a statistically significant correlation with tumour size (*ρ*=0.27, *P*=0.004). The 20S proteasome serum levels were not associated with gender (*P*=0.1832) and N stage (*P*=0.2139), and did not correlate with age (*ρ*=0.08, *P*=0.3981). Patients with higher T stages tended to have higher 20S proteasome serum levels, although this difference did not reach statistical significance (*P*=0.0729).

### Association with DSS

**Figure 2:**
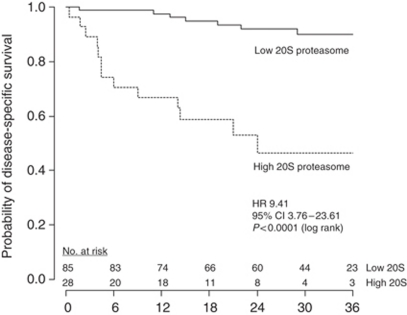
Association of 20S proteasome serum levels with DSS. Patients with high 20S proteasome serum levels had a 9.41-fold increased risk of death from CCRCC, compared with patients with low 20S proteasome serum levels. The optimal cut point of 7.24 *μ*g ml^−1^ was identified through recursive partitioning.

Univariable Cox proportional hazards analysis showed a hazard ratio of 1.21 (95% CI, 1.11–1.31), indicating that the risk of death from CCRCC increased by 21% by each 1 *μ*g ml^−1^ increase in 20S proteasome serum levels (*P*<0.0001). For graphical illustration with Kaplan–Meier survival estimates, a recursive partitioning-based survival tree analysis was carried out. A cut point of 7.24 *μ*g ml^−1^ was identified for further prognostic sub-stratification. Using this cut point, there were 85 patients (75%) with low levels and 28 (25%) with high levels. The 1- and 3-year survival probabilities (±s.e.) for patients with low levels *vs* high levels were 98±2% *vs* 67±9%, and 90±4% *vs* 46±11%, respectively (*P*<0.0001, Figure 2). In the multivariable model, SSIGN score and 20S proteasome serum levels were retained as independent prognostic factors (Table 2). The predictive accuracy of SSIGN score alone was 91.4%. It increased significantly to 92.5% (*P*=0.0025) with continuous, and to 93.2% (*P*=0.0030) with dichotomised 20S proteasome serum levels.

### Association with response to systemic therapy

In 24 patients with metastatic disease, a systemic first-line therapy was administered. Both patients, who had received temsirolimus, had progressive disease at first re-imaging and died from CCRCC after 4 and 5 months, respectively. Of the 22 patients, who had received sunitinib, there were 6 (27%) with progressive disease, 11 (50%) with stable disease, and 5 (23%) who exhibited a partial response. The 20S proteasome serum levels decreased with increasing response category (Table 3). However, due to the low numbers of patients, this difference did not reach statistical significance (*P*=0.2058).

## Discussion

This is the first study, which evaluated the putative significance of preoperative 20S proteasome serum levels in CCRCC. We found that this marker is elevated in patients with CCRCC, as compared with healthy controls. Further, a significant association with advanced tumour stage, tumour necrosis, and high Fuhrman grade was observed. The 20S proteasome serum levels were independently associated with DSS and increased the predictive accuracy of a standard prognostic model. Finally, this study generates the hypothesis that this marker may be helpful for the identification of patients with metastatic disease, who will most likely benefit from sunitinib treatment.

In our study, circulating 20S proteasome levels were elevated in patients with CCRCC. Lavabre-Bertrand *et al* (2001) were the first that assessed the role of this serum marker in cancer patients. Here, patients with solid tumours, myeloproliferative, and myelodysplastic syndromes had significantly higher levels than healthy donors. In the last years, similar data have been observed in other malignancies, including multiple myeloma (Jakob *et al*, 2007), breast cancer (Hoffmann *et al*, 2011), ovarian cancer (Heubner *et al*, 2011), and malignant melanoma (Stoebner *et al*, 2005). The source of this elevated 20S proteasome levels is unclear. It has been suspected that the proteasome levels originate from both tumour cells and non-malignant cells as a result of an immune reaction. Reinforcing the former concept, the proteasome is overexpressed in RCC and correlates with proliferation (Kanayama *et al*, 1991). This fact may also explain why 20S proteasome serum levels were associated with variables of tumour burden and differentiation, such as tumour size, M stage, and grade. In malignant melanoma, a similar relationship was observed (Stoebner *et al*, 2005). However, immunohistochemical expression in the tumour did not correlate with circulating proteasome levels in several studies (Heubner *et al*, 2011; Hoffmann *et al*, 2011), indicating a role of non-malignant cells and benign diseases. In fact, benign conditions, including vascular, pulmonary, or autoimmune diseases can significantly alter circulating proteasome levels (Egerer *et al*, 2002; Roth *et al*, 2005). In our pilot study, we did not investigate the relationship between immunohistochemical staining and circulating proteasome levels; however, this should be done in further studies.

Prediction of CCRCC prognosis and response to targeted therapy is mainly based on clinical and pathological variables. Conventional prognostic factors include performance status, TNM stage, grade, tumour size, and tumour necrosis. As one factor alone is not sufficient to predict prognosis accurately, multiple prognostic factors have been combined into prognostic models and nomograms (Frank *et al*, 2002; Zisman *et al*, 2002; Karakiewicz *et al*, 2007). For example, the SSIGN score combines TNM stage, size, grade, and necrosis for patients with CCRCC. Recent progress in molecular biology has identified novel genetic and protein markers, which may represent additional indicators of patients with biologically aggressive, high-risk RCC (de Martino *et al*, 2012). The current study identified 20S proteasome serum levels as such a prognostic biomarker. Several other small studies were thought to identify preoperative prognostic serum markers. Thompson *et al* (2008) studied preoperative serum levels of B7x in 101 patients with CCRCC. Serum levels were higher in patients with a tumour thrombus, positive lymph nodes, and distant metastases. In a study on 74 patients, preoperative VEGF-A levels were independently associated with poor DSS (Klatte *et al*, 2007a). Li *et al* (2008) showed that higher preoperative carbonic anhydrase-9 levels correlate with diminished recurrence-free survival. However, these markers were not validated externally and predictive accuracies were not assessed.

In this regard, the predictive accuracy of prognostic factors is generally assessed by the concordance index. Prognostic factors may be statistically significant in multivariable analysis, but it is possible that they do not add predictive information and may be therefore less relevant. Previous studies showed a predictive accuracy of the SSIGN score of 82% and 90%, respectively (Frank *et al*, 2002; Ficarra *et al*, 2006), which is in accordance with the current report. Of utmost importance, the predictive accuracy of the SSIGN score increased significantly when 20S proteasome serum levels were introduced as a variable. If validated by other groups, 20S proteasome serum levels may assist in postoperative risk stratification and may therefore allow risk-adjusted follow-up, or identify patients that should be included in adjuvant clinical trials.

It is similarly crucial to predict response to systemic therapy in patients with metastatic disease; however, this ability is limited by conventional prognostic models. In many patients, sunitinib will represent the first-line therapy. Attempts have been made to identify molecular markers predicting response to sunitinib. Paule *et al* (2010) evaluated the expression of 16 biomarkers in the primary tumour and identified soluble VEGF isoforms as predictors of response to sunitinib. Our data on 22 patients receiving sunitinib generate the hypothesis that circulating 20S proteasome serum levels may be another predictive marker. We analysed pre-operative samples, but not the levels following surgery, and thus, before the start of sunitinib. However, this should be done in a future study testing this hypothesis. The 20S proteasome serum levels may be further tested as a marker predicting response to bortezomib, as bortezomib reversibly inhibits the 20S proteasome subunit. Of 37 patients with metastatic RCC that were evaluated in a phase II trial, 4 (11%) achieved a partial response and 11 (38%) stable disease (Kondagunta *et al*, 2004). This clinical benefit rate may increase with proper patient selection according to the 20S proteasome serum levels.

Study limitations have to be acknowledged and addressed. As a single centre pilot study, few patients with limited follow-up were included. Sub-group analysis, for example, from patients receiving sunitinib therapy was limited by sample size. However, specific sub-group analyses were beyond the scope of this study. An external validation on large, prospective, multi-institutional cohorts with long-term follow-up will be necessary before this marker can be applied in clinical routine. The hypotheses generated, such as the role as a predictive marker for targeted therapy, deserve further investigation.

In summary, we identified circulating 20S proteasome as a novel diagnostic and prognostic serum marker for CCRCC. Its routine use may allow better diagnosis, risk stratification, risk-adjusted follow-up, and identification of patients with a greater likelihood of response to sunitinib therapy. External, prospective validation on large cohorts is warranted.

## Figures and Tables

**Table 1:** Descriptive statistics of CCRCC patients and associations with 20S proteasome serum levels

	**RCC population**		**20S proteasome levels**		
**Variable**	**No.**	**%**	**Median**	**IQR**	***P*-value**
*Gender*					0.1832
Female	38	34	5.40	4.54	—
Male	75	66	4.30	4.27	—
					
*Symptoms*					0.0008
No	85	75	4.03	3.69	—
Yes	28	25	7.40	7.51	—
					
*T stage*					0.0729
pT1–2	54	48	4.00	4.19	—
pT3	59	52	5.17	4.27	—
					
*N stage*					0.2139
pNx/N0	109	96	4.65	4.19	—
pN1	4	4	7.04	2.84	—
					
*M stage*					0.0011
M0	90	80	4.21	3.91	—
M1	23	20	7.43	7.05	—
					
*Fuhrman grade*					0.0247
Grade 1–2	82	73	4.29	4.01	—
Grade 3–4	31	27	6.20	6.11	—
					
*Tumour necrosis*					0.0462
No	61	54	3.98	4.05	—
Yes	52	46	5.27	4.20	—
					
*SSIGN*					0.0050
0–2	51	45	3.88	4.03	—
3–7	40	35	4.65	3.47	—
8 or greater	22	19	7.39	7.04	—

Abbreviations: CCRCC=clear cell renal cell carcinoma; IQR=interquartile range: SSIGN=T, N, M stage, size, grade, and necrosis.

**Table 2:** Multivariable Cox proportional hazards models

	**HR**	**95% CI**	***P*-value**	**C-index (%)**
*Model 1*				
SSIGN score	1.69	1.39–2.06	<0.0001	92.5
20S proteasome continuous	1.17	1.06–1.28	0.0015	
				
*Model 2*				
SSIGN score	1.60	1.33–1.92	<0.0001	93.2
20S proteasome categorical	4.03	1.55–10.44	0.0041	

Abbreviations: CI=confidence intervals; C-index=concordance index; HR=hazards ratio; SSIGN=T, N, M stage, size, grade, and necrosis.

The models included SSIGN score and 20S proteasome serum level as continuous (model 1) or categorical variable (model 2). In both models, 20S proteasome serum levels were retained as independent prognostic factor. The C-index increased significantly from 91.4 to 92.5% and 93.2%, respectively, after the 20S proteasome serum level was introduced in the model.

**Table 3:** 20S proteasome serum levels decreased with increasing response category

			**20S proteasome levels**	
**Response category**	**No.**	**%**	**Median**	**IQR**
Progressive disease	6	27	8.18	7.40
Stable disease	11	50	6.20	3.80
Partial response	5	23	3.65	0.84

Abbreviation: IQR=interquartile range.
